# 大细胞肺癌小肠转移1例病例报告和文献复习

**DOI:** 10.3779/j.issn.1009-3419.2010.06.019

**Published:** 2010-06-20

**Authors:** 燕伟 刘, 丽芝 张, 晓雨 韩, 涛 周

**Affiliations:** 1 116011 大连，大连医科大学 Dalian Medical University, Dalian 116011, China; 2 116011 大连，大连医科大学附属第一医院病理科 Department of Pathology, First Affiliated Hospital of Dalian Medical University, Dalian 116011, China; 3 116011 大连，大连医科大学附属第一医院肿瘤内科 Department of Medical Oncology, First Affiliated Hospital of Dalian Medical University, Dalian 116011, China

大细胞肺癌占肺癌的10%-15%，是非小细胞肺癌的相对少见病理类型，其组织分化差，易发生转移，小肠转移在临床很少见，其诊断多在出现梗阻或穿孔时，现报道1例大连医科大学第一附属医院肿瘤内科收治的大细胞肺癌小肠转移病例。

## 1 临床资料

患者郑xx，男性，67岁。2009年1月15日以“胸闷、气短15天”为主诉入院。患者饮食欠佳，大便时有不成形，小便正常，近3个月体重下降约1.5 kg。既往自述曾患肺结核，未系统治疗，后复查已钙化。吸烟30年，约20支/天，已戒烟3年。过敏史及家族史均无特殊。专科查体右下肺叩诊略浊，听诊呼吸音略弱，未闻及干湿啰音。辅助检查：2009年1月13日外院CT示右侧胸腔积液，2009年1月3次胸水涂片检查见大量淋巴细胞及退变间皮细胞背景下少许异性细胞，外院门诊脱落细胞检查结果提示可见中分化腺癌细胞，诊断为右肺癌（CT4N2M1）Ⅳ期、右胸膜转移癌、右胸腔恶性积液、肺转移癌（图 1）。给予TP方案（多西他赛+顺铂）化疗1周期。2009年2月外院行肺肿块粗针穿刺组织病理检查（病理号：G41846）提示大细胞肺癌（图 2），予顺铂胸腔局部化疗、香菇多糖胸腔局部生物治疗。2009年3月2日予恩度联合多西他赛化疗，出现Ⅱ度骨髓抑制，对症治疗后好转，2009年3月复查CT，疗效评价为疾病进展（progressive disease, PD），予二线培美曲塞单药化疗2周期，2009年6月复查CT出现骨转移、肝转移（图 3），疗效评价为PD，行局部姑息放疗止痛，自服中药抗肿瘤治疗。

**1:**
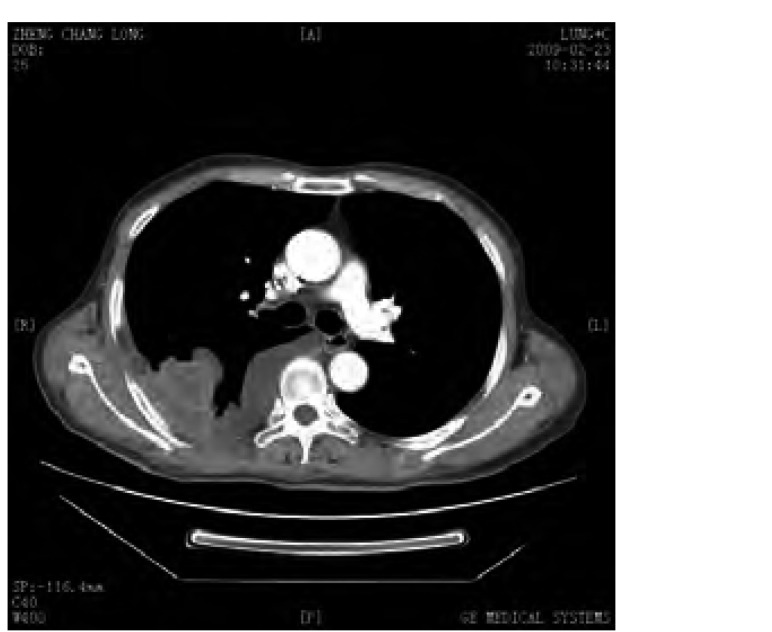
患者确诊时的CT显像。右肺原发灶，右胸膜转移癌，右胸腔积液 CT image of definite diagnosis. Right lung cancer, Right pleural metastasis, and Right pleural effusion

**2:**
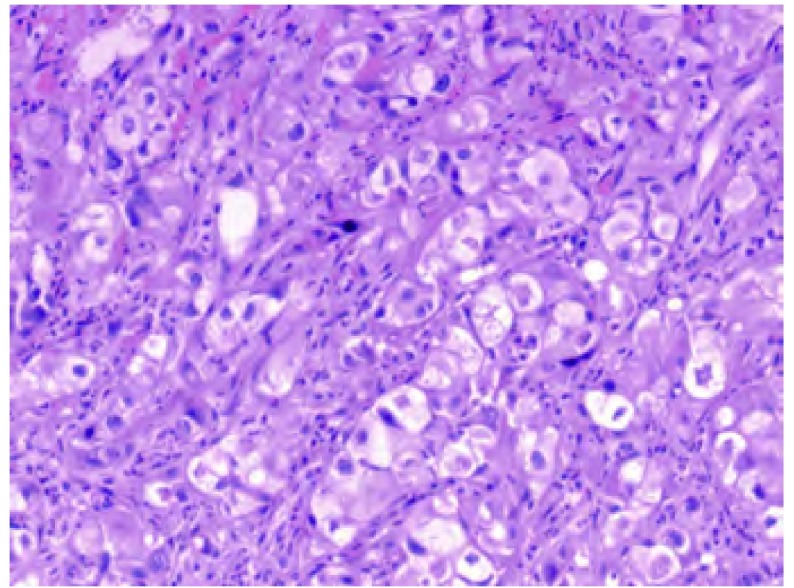
肺原发癌病理切片（HE，×100） Pimary lung cancer pathological section (HE, ×100)

**3:**
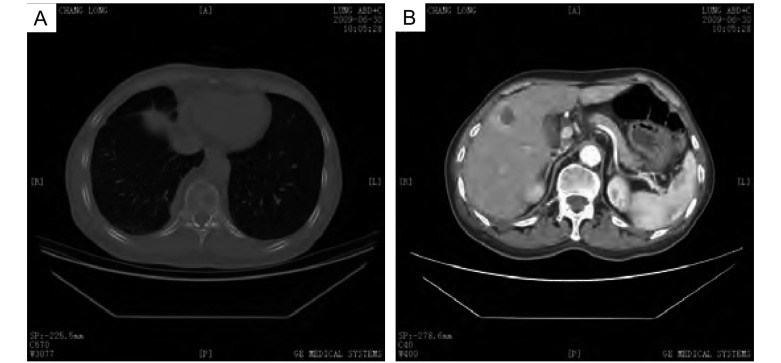
转移癌的CT显像。A：骨转移癌，B：肝转移癌 CT image of metastases. A: bone metastasis; B: hepatic metastasis

2009年8月10日因“停止排气排便，伴腹部胀痛呕吐3天”为主诉急诊入我院普外科，专科检查：腹部可见肠型，压痛（+），肠鸣音10次/分，可闻及气过水音，立位腹平片提示：小肠梗阻征象；CT提示：肝脏转移癌，盆腔小肠套叠伴低位小肠不全梗阻。诊断为小肠低位梗阻、小肠-小肠肠套叠、小肠肿瘤或小肠转移癌。于2009年8月11日行急诊手术，术中探查腹腔见浆液性腹水约2 000 mL，腹膜光滑，肝表面凹凸不平，触及转移结节，盆腔、肠系膜根部及腹主动脉旁未见转移灶，小肠扩张明显，肠壁水肿增厚，结肠无扩张。距屈氏韧带40 cm处的小肠系膜缘见肿物1枚，约1.5 cm×1.5 cm，外生性生长，考虑种植转移。距盲肠40 cm处回肠见小肠-小肠套叠，长度约10 cm，套叠肠管内可触及肿物，约2.5 cm×2.5 cm，质硬，肠管无缺血改变，套叠远处无扩张，结直肠未及肿物。行空回肠部分切除术，切除空肠约3 cm、回肠约20 cm，剖检肿物球形无蒂，未侵透肠壁，系膜缘肿物未侵及肠内壁，小肠及其肿物送病理检查，结果提示小肠见大细胞未分化癌，小肠两断端未见癌累及，肠系膜淋巴结未见癌转移0/2，另见癌结节2枚（病理号200913242）；免疫组化CK（+），TTF-1（-），CK7（+），CK20（-），考虑大细胞肺癌小肠转移（图 4）。

**4:**
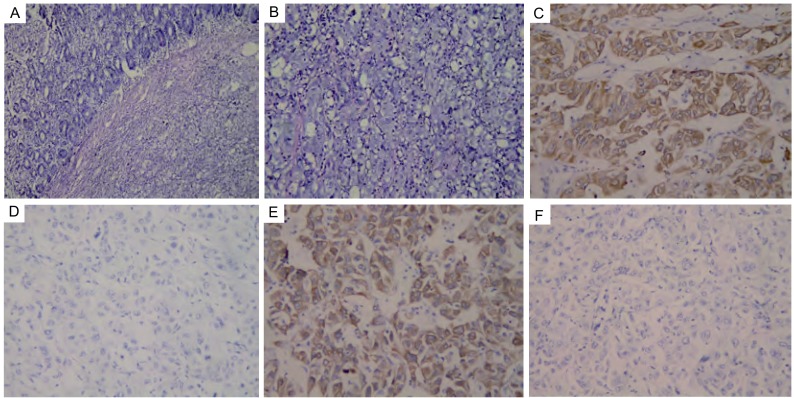
病理切片及免疫组化结果。A：小肠肿块（HE, ×100）；B：小肠肿块（HE, ×200）；C：CK（+）（×200）；D：TTF-1（-）（×200）；E：CK7（+）（×200）；F：CK20（-）（×200） Pathological section and immunohistochemisty. A: small intestine mass (HE, ×100); B: small intestine mass (HE, ×200); C: CK(+) (×200); D: TTF -1(-) (×200); E: CK7(+) (×200); F: CK20(-), (×200)

## 2 讨论

大细胞肺癌是肺癌组织学分型中非小细胞肺癌的一种，其组织分化比鳞癌、腺癌差，光镜下无腺样或鳞癌表现。临床上常表现为肺外周大肿块、侵犯亚段支气管或更大气道，但同腺癌相似易于出现区域淋巴结和远处转移。远处转移临床常见于脑、骨、肝、肾上腺、淋巴结^[1]^。复习国内文献资料，查得2000年以来国内共计报道14例非小细胞肺癌转移至胃肠道，其中6例由腺癌转移至直肠（1例）^[2]^及小肠（5例）^[3-7]^，4例由鳞癌转移至小肠（1例）^[8]^及结肠（3例）^[9-11]^，4例大细胞肺癌转移至小肠^[12-14]^。

小肠转移癌早期诊断困难，当肺癌患者出现一些非特异性的症状如腹胀、腹痛、腹泻以及进行性的贫血等症状时，尤其已有腹腔脏器（如肝、肾上腺、淋巴结）转移时，应怀疑有胃肠道转移的可能，临床实验室检查及内镜、CT、FDG PET/CT等技术对小肠转移癌的早期诊断是必要的^[15]^。随着PET-CT在临床的逐渐开展，其在肺癌转移诊断中的优势渐渐突显，关于PET在早期肺癌的分期中的应用，Maziak等进行的一项随机对照临床试验^[16]^得出结论，PET-CT组比常规检查组（骨扫描加腹部CT）发现更多转移病灶，减少不必要的外科手术（14% vs 7%），能够更好地预示早期非小细胞肺癌患者的全身转移情况。但目前PET-CT对小肠转移癌的诊断价值尚不明确。病变进展后患者可出现肠梗阻或肠穿孔表现，常需急诊手术探查明确诊断。

本例患者为老年男性，大细胞肺癌诊断明确，经历了化疗、放疗及生物治疗等综合治疗，但最终的疗效不十分令人满意，后出现小肠梗阻行手术探查，术后病理及免疫组化支持大细胞肺癌转移癌。

原发性小肠肿瘤和小肠转移肿瘤的鉴别，应结合患者病史、体征及相关的辅助检查，如X线钡餐检查、CT、PET-CT等，最终诊断绝大多数需手术取病理检查。病理诊断除了行常规HE染色外，还应行免疫组化检查，若免疫组化提示退行性改变者特别是多发肿瘤，推荐作TTF-l、CK、CK7、CK20，其中TTF-1阳性有诊断意义。临床上肺癌的小肠转移癌很少见，多数是出现了小肠穿孔后急诊入院。小肠穿孔是严重影响患者死亡率的因素，国外有数据分析不同的肺癌类型导致小肠穿孔的几率不同，腺癌占23.7%，鳞癌占22.7%，大细胞癌占20.6%，小细胞癌占19.6%^[17]^。同时Tomas等^[18]^认为对于发现小肠肿瘤的患者，应该首先排除是否为大细胞肺癌转移而来，这种转移，比我们预想的要常见得多。大细胞肺癌预后较差，尤其是出现肠道转移时多说明已出现广泛转移，有报道建议每一个肺癌的急腹症患者都应该考虑是肠转移，因为小肠转移癌几乎没有或很少有临床症状，加之平时小肠的临床体检不易发现阳性体征，导致小肠转移多为尸检诊断^[19]^，其实如果能够早发现对患者的预后是有好处的，尤其对于平时身体状况较好，肺癌疾病本身控制较好的小肠转移癌患者手术后比发现晚控制不好的患者生存时间长^[20]^。
